# Pressure-support improves lung protection and reduces cardiovascular dysfunction compared to pressure-controlled ventilation in experimental emphysema

**DOI:** 10.1186/2197-425X-3-S1-A569

**Published:** 2015-10-01

**Authors:** G Padilha, I Henriques, L Moraes, LF Horta, C Praga, I Ramos, PJ Miranda, M de Oliveira, C Santos, R Goldenberg, V Capelozzi, P Pelosi, P Silva, P Rocco

**Affiliations:** Federal University of Rio de Janeiro, Rio de Janeiro, Brazil; University of Sao Paulo, São Paulo, Brazil; University of Genoa, Genoa, Italy

## Introduction

Acute exacerbations of pulmonary emphysema lead to increased morbidity, mortality and, in some cases, requirement of invasive mechanical ventilation (MV) [1]. So far, no study has compared the biological impact of pressure-controlled ventilation (PCV) versus pressure-support ventilation (PSV) in experimental emphysema. Our aims were to develop an elastase-induced emphysema model in rats, and to compare the effects of PCV and PSV on the lung and heart.

## Methods

Forty-eight Wistar rats were randomly divided into two groups. In the emphysema (E) group, rats received porcine pancreatic elastase (2 IU) intratracheally, once a week for 4 weeks, whereas control (C) animals were treated with saline. Lung mechanics and histology, as well as echocardiography, were analyzed at 4, 6, 8, and 11 weeks. An additional 28 animals were treated with saline (n = 14) and elastase (n = 14) using the same protocol previously described and randomized according to ventilator strategy: pressure-controlled ventilation (C-PCV and E-PCV, n = 7/each) or pressure-support ventilation (C-PSV and E-PSV, n = 7/each). Tidal volume (V_T_) was kept constant (» 6 ml/kg) during the 4 hours of MV. Lung mechanics and echocardiography were assessed at the beginning (Initial) and at 4 hours of MV (End), after which animals were euthanized and lungs removed for morphometric and molecular biology analysis.

## Results

Compared to C animals, E animals exhibited increased specific elastance, functional residual capacity, mean alveolar diameter (Lm), and right ventricle area at 6 and 8 weeks. At 11 weeks, no significant changes were observed in lung mechanics, but Lm and right ventricle area remained elevated. Based on these data, we chose to compare mechanical ventilation strategies at 8 weeks. Mean airway and transpulmonary (P_Lmean_) pressures as well as areas of hyperinflation were lower in E-PSV compared to E-PCV animals at End (p < 0.001; p < 0.001; p < 0.05). The ratio between the pulmonary artery acceleration time and the pulmonary artery ejection time was lower in E-PCV than E-PSV at End, suggesting increased arterial pulmonary hypertension. The expression of cytokine-induced neutrophil chemoattractant (CINC)-1 and amphiregulin expression was increased in E-PCV than E-PSV. P_Lmean_ was well correlated with CINC-1 (r = 0.72 and p = 0.005) and amphiregulin (r = 0.98 and p < 0.001) in E animals.

## Conclusion

In this model of elastase-induced emphysema, PSV titrated to reach protective tidal volume, compared to PCV, improved lung mechanics, reduced lung hyperinflation and cardiovascular dysfunction, as well as prevented ventilator induced lung injury.

## Grant Acknowledgment

CNPq, FAPERJ, CAPES, MS-DECIT

## References

[1] Chandra D, Stamm JA, Taylor B, Ramos RM, Satterwhite L, Krishnan JA (2012). Outcomes of noninvasive ventilation for acute exacerbations of chronic obstructive pulmonary disease in the United States, 1998-2008. Am J Respir Crit Care Med.

